# Potentially inappropriate prescribing among older people in the United Kingdom

**DOI:** 10.1186/1471-2318-14-72

**Published:** 2014-06-12

**Authors:** Marie C Bradley, Nicola Motterlini, Shivani Padmanabhan, Caitriona Cahir, Tim Williams, Tom Fahey, Carmel M Hughes

**Affiliations:** 1 Clinical and Translational Epidemiology Branch, National Cancer Institute, Rockville, MD, USA; 2 HRB Centre for Primary Care Research, Department of General Practice, Royal College of Surgeons in Ireland, Beaux Lane House, Mercer Street, Dublin, Ireland; 3 Department of Pharmacology and Therapeutics, Trinity College Dublin, Dublin, Ireland; 4 Clinical Practice Research Datalink, Medicines and Healthcare Products Regulatory Agency, London, UK; 5 Clinical and Translational Epidemiology Branch, Epidemiology and Genomics Research Program, Division of Cancer Control and Population Sciences, National Cancer Institute, 9609 Medical Center Drive, 4E320, 20850 Rockville, MD, USA

**Keywords:** Potentially inappropriate prescribing, Older people, Screening tool of older persons potentially inappropriate Prescriptions (STOPP), CPRD

## Abstract

**Background:**

Potentially inappropriate prescribing (PIP) in older people is associated with increases in morbidity, hospitalisation and mortality. The objective of this study was to estimate the prevalence of and factors associated with PIP, among those aged ≥70 years, in the United Kingdom, using a comprehensive set of prescribing indicators and comparing these to estimates obtained from a truncated set of the same indicators.

**Methods:**

A retrospective cross-sectional study was carried out in the UK Clinical Practice Research Datalink (CPRD), in 2007. Participants included those aged ≥ 70 years, in CPRD. Fifty-two PIP indicators from the Screening Tool of Older Persons Potentially Inappropriate Prescriptions (STOPP) criteria were applied to data on prescribed drugs and clinical diagnoses. Overall prevalence of PIP and prevalence according to individual STOPP criteria were estimated. The relationship between PIP and polypharmacy (≥4 medications), comorbidity, age, and gender was examined. A truncated, subset of 28 STOPP criteria that were used in two previous studies, were further applied to the data to facilitate comparison.

**Results:**

Using 52 indicators, the overall prevalence of PIP in the study population (n = 1,019,491) was 29%. The most common examples of PIP were therapeutic duplication (11.9%), followed by use of aspirin with no indication (11.3%) and inappropriate use of proton pump inhibitors (PPIs) (3.7%). PIP was strongly associated with polypharmacy (Odds Ratio 18.2, 95% Confidence Intervals, 18.0-18.4, P < 0.05). PIP was more common in those aged 70–74 years vs. 85 years or more and in males. Application of the smaller subset of the STOPP criteria resulted in a lower PIP prevalence at 14.9% (95% CIs 14.8-14.9%) (n = 151,598). The most common PIP issues identified with this subset were use of PPIs at maximum dose for > 8 weeks, NSAIDs for > 3 months, and use of long-term neuroleptics.

**Conclusions:**

PIP was prevalent in the UK and increased with polypharmacy. Application of the comprehensive set of STOPP criteria allowed more accurate estimation of PIP compared to the subset of criteria used in previous studies. These findings may provide a focus for targeted interventions to reduce PIP.

## Background

Appropriate medications in older people have a clear evidence-based indication, are well tolerated and are cost-effective. In contrast, medicines that are potentially inappropriate, lack evidence-based indications, pose a higher risk of adverse effects or are not cost-effective [1].

Appropriateness of prescribing in older people has been most extensively assessed by process measures (provider’s actions) [2]. Explicit process measures are criterion-based and indicate drugs to be avoided in older people, independent of diagnoses or in the presence of certain diagnoses [3-5]. Explicit measures, requiring little clinical detail, can often be applied to large prescribing databases [2].

The United States (US) Beers criteria, the most commonly used explicit process measure for assessing potentially inappropriate prescribing (PIP) in older people, has been widely validated [6,7], but has some limitations; for example, approximately 50% of the Beers drugs are unavailable in European countries [8]. The recently developed ‘Screening Tool of Older Persons potentially inappropriate Prescriptions’ (STOPP) provides a more comprehensive explicit process measure of PIP, is validated for use in European countries [9], and overcomes some of the limitations inherent in the Beers criteria. STOPP is a physiological system-based screening tool comprising 65 clinically significant criteria which take drug-drug and drug-disease interactions, drug doses and duration of treatment into consideration. It considers clinical effectiveness and the removal of any potentially unnecessary drugs as well as drug duplication.

Optimisation of drug prescribing for older people is essential due to the substantial clinical and economic implications of drug-induced illness. PIP in older people has been associated with significant morbidity, adverse drug events (ADEs), hospitalisation and mortality [10-13]. PIP prevalence rates in older people have ranged from 14% to 37% in the US and Canada respectively, 19.8% in Europe [14] and 28% in the United Kingdom (UK) using the Beers criteria [15]. Further studies of PIP in the UK using large representative national databases, to identify the most common national PIP issues have been called for [15].

Previous studies of PIP have been limited to using a truncated version of the STOPP criteria due to a lack of clinical data in the available databases [16,17]. These studies used prescribing databases to investigate PIP in Northern Ireland (NI) and the Republic of Ireland (ROI). However, failure to apply the more complete set of STOPP criteria may have led to an underestimation of PIP and failure to identify important instances of PIP. Using the Clinical Practice Research Datalink (CPRD), the world’s largest computerized database of anonymized longitudinal patient records from primary and secondary care, may overcome this problem. As CPRD provides a complete record of clinical and prescribing data, a more comprehensive set of criteria can be applied which may more accurately reflect PIP prevalence.

Therefore, the overall aim of this study was to estimate the prevalence of PIP, in older people, in the UK, by applying a comprehensive set of 52 of the European based STOPP criteria to the CPRD and then to compare this to estimates obtained from applying the truncated version of the criteria to the same data. We also sought to determine the effect of factors such as polypharmacy, age, sex and co-morbidity on the prevalence of PIP which other studies have reported to be significant [16,17].

## Methods

### Setting

CPRD data from 2007 were used to examine PIP among older people, in a cross sectional study design, in the UK, using 52 of the 65 STOPP criteria, which have been described previously [9]. The term ‘UK’ will be used to refer to the findings resulting from the CPRD database throughout this paper. As stated in the Background, CPRD is the world’s largest computerized database of anonymized longitudinal patient records from primary care. It collects data from around 660 general practices in the UK, covers about 8.5% of the population and is broadly representative in terms of age, sex and geography. As of March 2013, there were 12.6 million acceptable (research quality) patients, of which 5.4 million are active (alive and registered with a contributing general practice).

Demographic information, lifestyle data, prescription details, clinical events and diagnoses, preventive care, specialist referrals, and hospital admissions and their major outcomes are all recorded in the database [18]. Data comes from up-to-standard (UTS) general practices, described as those that meet pre-defined standards in terms of data quality and collection. The high quality of CPRD prescription and diagnosis information has been documented [19,20]. Ethical approval for all observational research using CPRD data has been obtained from a Multicenter Research Ethics Committee. Data were extracted in February 2012.

### Participants

The study population comprised all CPRD patients aged 70 years or older registered with an UTS practice during the study period 01/01/2007- 31/12/2007. All patients were required to have at least 3 months of lead-in data, prior to 01/01/2007, to ascertain long term use of certain medications. All data were anonymised and the research team had no access to any identifiable data.

### Exposures

Fifty two of the 65 STOPP indicators were deemed suitable for application to CPRD clinical and therapy data based on the available information. Some indicators could not be applied due to absence of certain types of clinical data. For example, “Long-term opiates in those with dementia unless indicated for palliative care or management of moderate/severe chronic pain syndrome” was difficult to ascertain and therefore, were not used. However, the availability of clinical as well as prescription information allowed a larger number of STOPP criteria to be applied than in previous studies [16,17]. Exposure status was based on prescription and clinical data in the database. Data on drug use were extracted using Multilex codes whilst clinical diagnoses were identified from Read codes. All codes were manually reviewed and confirmed by MB and an experienced primary care physician.

Patients were categorised into those who received a STOPP criteria drug or drug combination. STOPP criteria which specified a particular dosage not to be exceeded e.g. proton pump inhibitors (PPIs) at maximum therapeutic dosage for > 8 weeks, were evaluated by calculating the number of defined daily doses (DDDs) [21] for each recipient according to the DDD of the drug, and the strength and quantity of the dispensed medication for each prescription. A subset of 28 STOPP criteria which had been used in two previous investigations [16,17] were also applied to the data.

### Polypharmacy

The total number of prescriptions received for each different drug class was calculated for each participant, during the study period. A repeat medication was defined by receipt of 3 or more prescriptions for that agent in the study period. Polypharmacy was indicated by use of 4 or more repeat medications, each from different drug groups [22].

### Charlson comorbidity index

In order to investigate the potential effect of co-morbid conditions on PIP, we applied the Charlson comorbidity index (CCI) to the CPRD data. The CCI is the most widely studied morbidity index and its validity has been confirmed by comparison with other indices [23,24]. It has also been validated for application to longitudinal databases [25]. The CCI takes account of both the number and severity of the comorbid conditions.

### Outcomes

The main outcome was the overall prevalence of PIP in those aged ≥70 years in 2007 in the UK, according to the comprehensive set of 52 STOPP criteria and the subset of 28 criteria. Secondary outcome measures were: (i) the prevalence of PIP per individual STOPP criterion, and (ii) the association between PIP, polypharmacy, CCI, gender, and age group.

### Statistical analysis

The overall prevalence of PIP, the corresponding 95% Confidence intervals (CIs) and the prevalence per individual STOPP criterion were calculated. Logistic regression analyses were used to determine the association between any (*vs.* no) PIP and polypharmacy (categorized as no polypharmacy vs polypharmacy), CCI (categorized as 0, 1, 2, 3, 4 points assigned), age group (70 to 74 years, 75 to 80 years, 81 to 85 years, 85+ years), and gender. Adjusted odds ratios (OR) and 95% confidence intervals (CI) were calculated. Data extraction and analysis were performed using STATA Version 12 (Timberlake Consultants Ltd, London, UK).

## Results

1,019,491 persons, aged ≥ 70 years, identified in the CPRD, were eligible for inclusion in the study. More than 50% were female (592,045, 58%) and 78.5% (799,948) were aged ≥ 75 years as shown in Table 1.

**Table 1:** Descriptive characteristics of the study population in CPRD

	**PIP (n = 723,838)**	**No PIP (n = 295,653)**
**Gender**		
**-Male (%)**	122,817 (28.7)	304,622 (71.3)
**-Female (%)**	172,834 (29.2)	419,211 (70.8)
**-Missing (%)**	2	
**Age (years)**		
**-70**–**74 (%)**	82,177 (37.4)	137,366 (62.6)
**-75**–**80 (%)**	92,488 (37.6)	153,778 (62.4)
**-81**–**85 (%)**	62,407 (33.1)	126,040 (66.9)
**- > 85 (%)**	58,581 (18)	306,654 (84)
**Morbidities (Charlson morbidity index score)**		
**-1 (%)**	189,864 (28.3)	481,983 (71.7)
**-2 (%)**	52,365 (46.8)	59,519 (53.2)
**-3 (%)**	53,424 (22.7)	182,336 (77.3)
**Polypharmacy (≥4 medications)**		
**-Never (%)**	114,816 (14.6)	669,572 (85.3)
**-Ever (%)**	180,837 (76.9)	54,266 (23.1)
**Chronic Obructive Pulmonary Disease**		
**-No (%)**	277,497 (28.2)	707,447 (71.8)
**-Yes (%)**	18,156 (52.6)	16,391 (47.5)
**Peptic ulcer**		
**-No (%)**	274,487 (28.9)	675,938 (71.1)
**-Yes (%)**	21,166 (30.7)	47,900 (69.4)
**Diabetes**		
**-No (%)**	225,280 (27.3)	625,591 (72.7)
**-Yes (%)**	70,373 (41.7)	98,247 (58.3)
**Dementia**		
**-No (%)**	283,983 (28.5)	710,985 (71.5)
**-Yes (%)**	11,670 (47.6)	12,853 (52.4)
**Hypertension**		
**-No (%)**	140,467 (21.1)	525,316 (78.9)
**-Yes (%)**	155,186 (43.9)	198,522 (56.1)
**Osteoarthritis**		
**-No (%)**	216,981 (26.5)	601,325 (73.5)
**-Yes (%)**	78,672 (39.1)	122,513 (60.9)
**Heart failure**		
**-No (%)**	292,294 (29.0)	715,868 (71.0)
**-Yes (%)**	3,359 (29.7)	7,970 (70.4)
**Parkinsonism**		
**-No (%)**	290,071 (29.0)	709,721 (71.0)
**-Yes (%)**	5,582 (28.3)	14,117 (71.7)

### Main outcomes

#### Overall prevalence of PIP in the UK in 2007 using 52 STOPP criteria

The overall prevalence of PIP in the UK, according to the 52 STOPP indicators, was 29% (95%CIs 28- 29%) (n = 295,653). Just under 29% (28.7%) of males had PIP in the study period compared to 29.2% of females. Of those aged 70–74, 37.4% had a PIP compared to 16% of those aged > 85 years. (Table 1) Almost 15% of the population, (148,614 patients) were prescribed one potentially inappropriate medication, 77,923 (7.6%) were prescribed two and 69,116 (6.8%) were prescribed three or more.

#### Prevalence of PIP according to individual STOPP criteria

Table 2 describes the prevalence for each individual STOPP criteria, listed by physiological system. The most common issue of PIP was therapeutic duplication (121,668 patients 11.9%), followed by use of aspirin with no history of coronary, cerebral or peripheral vascular symptoms or occlusive arterial event (115,576 patients 11.3%). Use of PPIs at maximum therapeutic dose for > 8 weeks (38,153 patients, 3.7%) was the third most common PIP, whilst alpha blockers with long-term urinary catheter in situ (31,226 patients 3.1%) was next. Many other criteria had a prevalence less than 0.5%.

**Table 2:** Prevalence of potentially inappropriate prescribing by individual STOPP criteria among older people in CPRD

**Criteria description**	**Number of patients (N = 1,019,491)**	**% of patients (95% CIs)**
**Cardiovascular system**		
Digoxin > 125 mcg/day ***(increased risk of toxicity)***^***a***^	9327	0.9 (0.8-0.9)
Thiazide diuretics with gout ***(exacerbates gout)***	6094	0.6 (0.6-0.6)
Beta-blocker + verapamil ***(risk of symptomatic heart block)***	503	0.05 (0.05-0.05)
Aspirin + Warfarin without a PPI/ H_2_RA ***(high risk of gastrointestinal bleeding)***	3616	0.4 (0.3 -0.4)
Dipyridamole as monotherapy for cardiovascular secondary prevention ***(no evidence of efficacy)***	2137	0.2 (0.2-0.2)
Aspirin > 150 mg/day **(*increased bleeding risk)***	5128	0.5 (0.5-0.5)
Loop diuretic for dependent ankle oedema only i.e. no clinical signs of heart failure ***(no evidence of efficacy, compression hosiery usually more appropriate)***	25843	2.54 (2.5-2.6)
Loop diuretic as first-line monotherapy for hypertension ***(safer, more effective alternatives available)***	7128	0.7 (0.7-0.7)
Non-cardioselective beta-blocker with Chronic Obstructive Pulmonary Disease (COPD) ***(risk of bronchospasm)***	353	0.03 (0.03-0.03)
Calcium channel blockers with chronic constipation ***(may exacerbate constipation)***	16826	1.6 (1.6-1.7)
Aspirin with a past history of peptic ulcer disease without histamine H2 receptor antagonist or Proton Pump Inhibitor ***(risk of bleeding)***	3912	0.4 (0.4-0.4)
Aspirin with no history of coronary, cerebral or peripheral vascular symptoms or occlusive arterial event ***(not indicated)***	115576	11.3 (11.3-11.4)
**Central Nervous System**		
TCAs with dementia ***(worsening cognitive impairment)***	354	0.03 (0.03-0.03)
TCAs with glaucoma ***(exacerbate glaucoma)***	354	0.03 (0.03-0.03)
TCAs with opioid or calcium channel blocker ***(risk of severe constipation)***	26649	2.6 (2.6-2.6)
Long-term (>1 month) long-acting benzodiazepines ***(risk of prolonged sedation, confusion, impaired balance, falls)***	15057	1.5 (1.5-1.5)
Long-term (>1 month) neuroleptics (antipsychotics) **(*risk of confusion, hypotension, extrapyramidal side-effects, falls)***	21012	2.1 (2.1-2.1)
Long- term (>1 month) neuroleptics with parkinsonism ***(worsen extrapyramidal symptoms)***	852	0.1 (0.1-0.1)
Anticholinergics to treat extrapyramidal symptoms of neuroleptic medications ***(risk of anticholinergic toxicity)***	869	0.1 (0.1-1.0)
Phenothiazines with epilepsy ***(may lower seizure threshold)***	448	0.04 (0.04-0.04)
Prolonged use (>1 week) of first-generation anti-histamines ***(risk of sedation and anti-cholinergic side-effects)***	6020	0.6 (0.6-0.6)
TCA’s with cardiac conductive abnormalities	543	0.05 (0.05-0.05)
TCA’s with prostatism or prior history of urinary retention ***(risk of urinary retention)***	2623	0.3 (0.3-0.3)
TCA’s with constipation ***(likely to worsen constipation)***	7279	0.7 (0.7-0.7)
**Gastrointestinal System**		
Prochlorperazine or metoclopramide with parkinsonism ***(risk of exacerbating parkinsonism)***	385	0.04 (0.04)
PPI for peptic ulcer disease at maximum therapeutic dosage for > 8 weeks ***(dose reduction or earlier discontinuation indicated)***	38153	3.7 (3.7-3.8)
Anticholinergic antispasmodic drugs with chronic constipation ***(risk of exacerbation of constipation)***	1208	0.1 (0.1-0.1)
**Respiratory system**		
Systemic corticosteroids instead of inhaled corticosteroids for maintenance therapy in moderate-severe COPD ***(unnecessary exposure to long-term side-effects of systemic steroids)***	1339	0.1 (0.1-0.1)
Nebulised ipatropium with glaucoma *(****exacerbate glaucoma****)*	20	0
**Musculoskeletal system**		
Long term NSAID use (>3 months) with osteoarthritis ***(simple analgesics preferable)***	12167	1.2 (1.2-1.2)
Warfarin and NSAID use ***(risk of gastrointestinal bleeding)***	2495	0.2 (0.2-0.3)
Non-steroidal anti-inflammatory drug (NSAID) with history of peptic ulcer disease or gastrointestinal bleeding, unless with concurrent histamine H2 receptor antagonist, PPI or misoprostol ***(risk of peptic ulcer relapse)***	1040	0.1 (0.1-0.1)
NSAID with heart failure ***(risk of exacerbation of heart failure)***	409	0.04 (0.04-0.04)
NSAID with chronic renal failure ***(risk of deterioration in renal function)***	928	0.1 (0.1-0.1)
Long-term corticosteroids (>3 months) as monotherapy for rheumatoid arthrtitis or osteoarthritis ***(risk of major systemic corticosteroid side-effects)***	718	0.1 (0.1-0.1)
Long-term NSAID or colchicine for chronic treatment of gout where there is no contraindication to allopurinol ***(allopurinol first choice prophylactic drug in gout)***	2845	0.3 (0.3-0.3)
**Urinary System**		
Antimuscarinic drugs (urinary) with dementia ***(risk of increased confusion and agitation)***	297	0.03 (0.03-0.03)
Antimuscarinic drugs with chronic glaucoma ***(risk of acute exacerbation of glaucoma)***	109	0.01 (0.01-0.01)
Bladder antimuscarinic drugs with chronic constipation ***(risk of exacerbation of constipation)***	3514	0.3 (0.3-0.4)
Bladder antimuscarinic drugs with chronic prostatism ***(risk of urinary retention)***	2791	0.3 (0.3-0.3)
Alpha-blockers in males with frequent incontinence i.e. one or more episodes of incontinence daily ***(risk of urinary frequency and worsening of incontinence)***	1426	0.1 (0.1-0.2)
Alpha-blockers with long-term urinary catheter in situ i.e. more than 2 months ***(drug not indicated)***	31226	3.1 (3.0-3.1)
**Endocrine system**		
Beta-blockers in those with diabetes mellitus and frequent hypoglycaemic episodes ***(risk of masking hypoglycaemic symptoms)***	26563	2.6 (2.6-2.6)
Glibenclamide with type 2 diabetes mellitus ***(risk of prolonged hypoglycaemia)***	981	0.1 (0.1-0.1)
H. Drugs that adversely affect those prone to falls (≥1 fall in past three months)		0.3 (0.3-0.3)
1. Benzodiazepines ***(sedative, may cause reduced sensorium, impair balance)***	3358	0.2 (0.2-0.3)
2. Neuroleptic drugs ***(may cause gait dyspraxia, Parkinsonism)***	2491	
3. Firstgeneration antihistamines ***(sedative, may impair sensorium)***	250	0.02 (0.02-0.02)
4. Vasodilator drugs ***(postural hypotension)***	788	0.1 (0.1-0.1)
5. Long-term opiates in those with recurrent falls	10321	1.0 (0.1-1.0)
Two concurrent drugs from the same group- therapeutic duplication ***(optimization of monotherapy within a single drug class)***	121668	11.9 (11.9-12.0)

^a^Italised text in brackets represents the potential risk associated with the PIP indicators.

There was strong evidence of an association between PIP and polypharmacy. Those receiving 4 or more repeat medications were 18 times more likely to be exposed to PIP compared to those on 0–3 medications (OR 18.2, 95% CI, 18.0-18.4, P < 0.05). The odds of having a PIP was only slightly lower in females compared to males when adjusting for other factors (OR 0.9 95% CI 0.9- 0.9, P < 0.05). PIP was less common in those aged 85 years and above compared to those aged 70–74 years (OR 0.5, 95% CI, 0.4-0.5, P < 0.05). PIP was more common in those with fewer co-morbid conditions according to the CCI (Table 3).

**Table 3:** Unadjusted and adjusted ORs for the association between PIP and its predictors

**PIP (ever/never)**	**Unadjusted odds ratios (95% CIs)**	Adjusted odds ratios* and (95% CIs)
Polypharmacy		
-Never (ref)	1.0	1.0
-Ever	19.4 (19.2-19.7)	18.2 (18.0-18.4)
Age (years)		
-70–74 (ref)	1.0	1.0
-75–80	1.0 (1.0- 1.0)	0.9 (0.9-0.9)
-81–85	0.8 (0.8-0 .8)	0.8 (0.8-0.8)
- > 85	0.3 (0.3-0.3)	0.4 (0.4-0.4)
Gender		
-Male (ref)	1.0	1.0
-Female	1.0 (1.0-1.0)	0.9 (0.9- 0.9)
-Missing		1.5 (1.5-1.5)
Mobidities (Charlson morbidity index score)		
-1 (ref)	1.0	1.0
-2	2.2 (2.2-2.3)	1.51 (1.5-1.5)
-3	0.4 (0.4-0.40)	0.9 (0.9-0.9)

*Adjusted for age (70–74, 75–80, 81–85,>85 years), gender, morbidity (charlson morbidity index: 1 representing a lower number of comorbidities and 3 higher) and polypharmacy (ever/never).

#### Prevalence of PIP using 28 STOPP criteria

The prevalence of PIP in the UK was 14.9% (95% CIs 14.8-14.9%) (n = 151,598) when the subset of 28 STOPP indicators was applied. Just under 11% (109,808 patients) were in receipt of at least one case of PIP, whilst 3.1% (31,693 patients) were exposed to 2 or more instances and 1.0% (10,095 patients) were exposed to three or more. The most common PIP issues were use of PPIs at maximum therapeutic dose for > 8 weeks (3.7%, 38,153 patients), NSAIDs for > 3 months (3.2% 32,373 patients), and use of long-term neuroleptics (2.1%, 21,012 patients.

## Discussion

Following the application of 52 STOPP indicators to CPRD, the overall PIP prevalence, in those aged ≥ 70 years, in the UK, was estimated at 29%. The most common cases of PIP were therapeutic duplication, use of aspirin with no valid indication and inappropriate use of PPIs. PIP was associated with polypharmacy and was less common among those 85 years and above compared to younger age groups. It was also slightly more common in men. When a subset of 28 STOPP criteria, commonly used in other studies, were applied, the overall PIP prevalence for the UK was 14.9%. The most common instances of PIP on application of the subset were PPI use at maximum dose for greater than 8 weeks and the use of NSAIDs for > 3 months. Application of the 52 STOPP indicators in CPRD enabled a more comprehensive estimation of PIP and highlighted additional PIP issues that were not observed with the truncated version of the criteria.

### PIP in the UK (application of 52 indicators)

Therapeutic duplication and inappropriate use of aspirin with no valid indication were the most prevalent cases of PIP in the UK and have also been reported as prevalent among older hospitalised patients in Ireland [13]. Therapeutic duplication is difficult to conclusively identify in a medical record database as drugs, which have been switched within a therapeutic group, may appear on the medical record for a number of months following changes, even though they are not dispensed. The practice of prescribing aspirin to asymptomatic individuals for the prevention of myocardial infarction is common and may have influenced these findings. However, this practice has been questioned after a meta-analysis on the subject reported no benefit [26,27]. Inappropriate use of PPIs has been reported previously and targeting such use is critical to reducing the burden of PIP in older people [28-30].

The strong association between PIP and polypharmacy seen in this study has been reported elsewhere and the literature is replete with studies consistently demonstrating this association [31-34]. Polypharmacy is a common phenomenon in older adults, and whilst targeting polypharmacy represents an obvious approach to reducing PIP, the distinction between appropriate and inappropriate polypharmacy is not clearly defined [22]. One study demonstrated that despite rises in polypharmacy in the UK, largely suspected to be associated with better chronic disease management, no subsequent increase in PIP was seen, indicating that prescribing more medications does not always translate to a rise in PIP [15]. In this era of increased focus on chronic disease management and multi-morbidities, this is an on-going challenge for those responsible for prescribing in primary care.

This study revealed that PIP was less common as patients aged and this has also been widely documented [35,36]. Greater physician awareness of PIP in the oldest old and the higher mortality rate in this age group, as well as changing clinical priorities at the end of life have been postulated as potential explanations [37]. In this study, PIP was less likely in those with a higher score on the CCI compared to lower scores. This may also be related to advancing age as those who are older receive an additional rating on the CCI.

### PIP in the UK (application of 28 indicators)

As expected, application of the smaller subset of STOPP criteria to the CPRD data resulted in a lower prevalence of PIP. However, some of the most common instances of PIP differed from those identified using the larger set of criteria. As seen in previous studies [16,17], using this subset of criteria, tended to limit the investigation of PIP and may result in a failure to target important areas of prescribing that need attention in order to reduce the overall problem. The previous studies which applied this subset of criteria investigated PIP in NI and ROI [16,17]. Compared to those studies, the UK had a much lower overall prevalence of PIP (14.9%) [NI (34%) [16] and ROI (36%)] [17]. The number of patients in receipt of 2 or more instances of PIP was also lower in the UK compared to NI and ROI. The PPI and NSAIDs indicators were the most common for all three jurisdictions, however, there were marked differences in prevalence, notably in the PPI indicator. The comparative prevalence rates were 16.69% in ROI, 10.79% in NI and 3.74% in the UK.

NI has a similar healthcare system to the rest of the UK, yet the overall prevalence of PIP in NI was more similar to that reported in ROI, despite differences in their respective healthcare systems. Other studies that compared prescribing in the NI and ROI have reported commonalities [38]. The prevalence of certain criteria (use of long-term long-acting benzodiazepines) was high in NI and ROI (6.1% and 5.2% respectively) [16,17], yet much lower in the UK using the CPRD data (1.5%). Intensive prescribing initiatives in parts of the UK (excluding NI), as early as 1988 [39], to reduce inappropriate benzodiazepine prescribing, may have accounted for these differences and benzodiazepine dispensing decreased by 51.3% between 1980 and 2009, in England alone [40]. It has been suggested that the legacy of civil disturbances in NI, from previous decades, may have influenced patterns of benzodiazepine prescribing in this jurisdiction [41]. This highlights the multitude of factors influencing PIP, many of which may be difficult to modify.

The differences in PIP between regions may have been influenced by region-specific regulatory measures, as referred to in relation to benzodiazepines above. It has been suggested that implementation of prescribing guidelines and audits by clinical pharmacists may have contributed to the lower prevalence of PIP observed in the UK [14]. One study, which investigated PIP in nursing home residents across eight European countries, found a strikingly low PIP prevalence in Denmark compared to other European countries, despite high rates of polypharmacy [14].This low level was linked to the provision of a drug utilization review by the National Institute of Health, which included feedback to individual physicians. This raises the question of whether a more concerted effort between neighboring regions in developing policies to tackle PIP might be useful.

### Strengths and limitations

This is the largest study to date to investigate PIP in the UK. Prospectively collected prescription and clinical data from the CPRD, as well as accurate dosing information increased the reliability of the findings compared to previous studies. The availability of clinical data allowed more complete assessment of PIP. The use of a large national database gave a clear insight into the more common issues in PIP nationally rather than the local focus of some previous studies [15].

The STOPP criteria were designed for application in primary care settings with easy access to the patient’s full medical record. Despite the comprehensive patient information in CPRD, not all of the STOPP criteria could be applied. Failure to apply the full criteria may have resulted in overestimation of PIP in these instances.

In contrast, CPRD is a widely used and validated database with reliable prescription and clinical information collected from UTS practices across the UK. Whilst CPRD is representative of the UK population, the generalisability of the data may be limited by the fact that those practices that contribute to the database, meet pre-defined data and record-keeping quality standards. It is possible that such practices might also deliver enhanced quality prescribing which is less likely to be inappropriate compared to an average non-CPRD practice.

Identification of Read codes for clinical diagnoses was often ambiguous. This may have led to over- or under-estimation of the prevalence of some criteria. In order to reduce this potential misclassification, we sought the assistance of an experienced primary care physician who reviewed the codes. Therapeutic duplication, the most common example of PIP in this study, was difficult to accurately assess using medical record or prescription databases and may have been misrepresented. Whilst we attempted to account for such misrepresentation, it is still possible that therapeutic duplication was overestimated. Some patients may have belonged to practices that were inactive, or had transferred out of CPRD resulting in some data some loss during the study period. This could have potentially led to a slight underestimation of PIP.

## Conclusions

PIP is prevalent among older people across the UK, and is more accurately estimated by applying a comprehensive set of STOPP criteria to databases such as CPRD, compared to the truncated version used in previous studies, on more limited databases. However, comparison with previously published studies which had used a subset of the full STOPP criteria showed examples of PIP were consistent. Indicators such as the STOPP criteria and the newly updated Beers criteria [42] have their place in determining the presence of PIP and informing interventions to reduce the problem. However, it appears that more integrated approaches are needed to significantly reduce the burden of PIP. Previously suggested approaches in the UK have included identifying the main PIP issues nationally (which this study fulfilled) and the use of alert systems in the computers of primary care physicians to identify PIP at the time of prescribing [43]. Such systems have effectively reduced the level of newly prescribed inappropriate medications in the US [44] and similar pharmacist-led information technology interventions in the UK reduced medication errors in primary care, indicating the potential for future development [45]. It would appear from this study and previous findings [16,17] that there is a need for targeted interventions to reduce PIP across all regions but especially in NI and ROI. Targeted interventions focus on specific instances of PIP. The UK has, in the past, successfully introduced incentives to reduce inappropriate prescribing of particular drug groups such as benzodiazepines and these appear to have been successful in reducing the overall burden of PIP. The introduction of national guidelines on the prescribing of co-proxamol successfully led to reductions in the use of this preparation, resulting in its eventual discontinuation [46]. Such targeted interventions may provide a template for action in the other regions where PIP is higher and for some of the more common examples such as inappropriate use of PPIs. Polypharmacy appears to be a major influence on PIP, although attempts to reduce polypharmacy may prove challenging due to the current emphasis on chronic disease management in primary care.

## Competing interests

None of the authors have any conflicts of interest that need to be declared.

## Authors’ contributions

Conception and design: CMH, TF, MCB, CC. Acquisition of data: SP, TW, MCB, CMH, CC. Analysis and interpretation: MCB, SP, NM, CMH. Drafting of manuscript: MCB, CMH. Critical revision of the manuscript: MCB, CMH, TF. Obtaining funding: TF, CMH. All authors read and approved the final manuscript.

## Pre-publication history

The pre-publication history for this paper can be accessed here:

http://www.biomedcentral.com/1471-2318/14/72/prepub
